# The Role of Cell Division Autoantigen 1 (CDA1) in Renal Fibrosis of Diabetic Nephropathy

**DOI:** 10.1155/2021/6651075

**Published:** 2021-04-28

**Authors:** LinLin Chen, Jiao Wu, Bin Hu, Changbai Liu, Hu Wang

**Affiliations:** ^1^Affiliated Ren He Hospital of China Three Gorges University, Yichang 443002, China; ^2^Medical School, China Three Gorges University, 8 Daxue Road, Yichang 443002, China; ^3^Hubei Key Laboratory of Tumor Microenvironment and Immunotherapy, China Three Gorges University, 8 Daxue Road, Yichang 443002, China; ^4^Institute for Cell Engineering, Johns Hopkins University School of Medicine, Baltimore, MD 21205, USA

## Abstract

The common kidney disease diabetic nephropathy (DN) accounts for significant morbidity and mortality in patients with diabetes, and its effective diagnosis in incipient stages is still lacking. Renal fibrosis is the main pathological feature of DN. Cell division autoantigen 1 (CDA1), a phosphorylated protein encoded by TSPYL2 on the X chromosome, plays a fibrogenic role by modulating the transforming growth factor-*β* (TGF-*β*) signaling, but the exact mechanism remains unclear. TGF-*β* signaling has been recognized as the key factor in promoting the development and progression of DN. At present, strict control of blood sugar and blood pressure can significantly lower the development and progression of DN in the early stages, and many studies have shown that blocking TGF-*β* signaling can delay the progress of DN. However, TGF-*β* is a multifunctional cytokine. Its direct intervention may result in increased side effects. Therefore, the targeted intervention of CDA1 not only can block the TGF-*β* signaling pathway but also can reduce these side effects. In this article, we review the main physiological roles of CDA1, with particular attention to its effect and potential mechanism in the renal fibrosis of DN.

## 1. Introduction

Diabetes is one of the most important noncommunicable diseases that seriously threaten human health at present. Its prevalence rate is increasing year by year, and it has reached epidemic proportions worldwide. The rate of kidney disease diabetic nephropathy (DN), one microvascular complication of diabetes, continues to increase with the growing incidence of diabetes [1–3]. DN has become one of the main causes of end-stage renal disease (ESRD) and the death of diabetic patients [4]. It also lacks effective biological diagnostic biomarkers in the early stage. Microalbuminuria is the earliest recognized and noninvasive diagnostic indicator of DN. However, it is unable to be used as a marker for DN diagnosis in young patients and nonalbuminuric DN or to predict the risk from advanced stages in the progression of chronic kidney disease (CKD) [5]. DN is usually diagnosed too late and irreversible, meaning it cannot be effectively treated. Currently, the main treatment in the early stage of DN is too tightly control blood sugar, blood lipids, and blood pressure, as well as practicing lifestyle changes [6, 7]. Although some reports suggest that renin-angiotensin-aldosterone system inhibitors (RAASi) [8, 9], sodium-glucose cotransporter 2 inhibitors (SGLT-2i) [10–12], glucagon-like peptide-1 receptor agonists (GLP-1 RA) [10, 13, 14], and endothelin receptor antagonists (ERAs) [15–17] have renoprotective properties in delaying the progression of DN, they cannot stop DN progression to end-stage renal failure. This is because of the persistent secondary pathological processes [18]. Therefore, it is necessary to develop new treatments to delay the occurrence and development of DN.

The pathogenesis of DN is complex and multifactorial. Renal fibrosis is the final pathological change in DN [19], characterized by the accumulation of extracellular matrix (ECM) [20]. TGF-*β* is considered to be a core pathway leading to renal fibrosis [21]. In various disease models, inhibition of TGF-*β*1 or its downstream signaling pathway significantly restricted renal fibrosis, whereas overexpression of TGF-*β*1 induces renal fibrosis [22]. Many studies have shown that blocking the TGF-*β* signaling pathway can delay the progression of DN. But TGF-*β*1, a member of the TGF-*β* superfamily, is a pleiotropic cytokine with a wide variety of physiological roles that include not only regulating tissue fibrosis but also regulating many biological responses, including wound healing [23], cell proliferation, cell differentiation, ECM production and remodeling, chemotaxis, growth factor and hormone production [24], angiogenesis and hematopoiesis [25], immune regulation [26], and cell apoptosis and autophagy [27]. Direct blocking of TGF-*β* signaling pathway may result in many other adverse events, such as prenatal lethality [28], excessive inflammatory response [29] and immune dysregulation [30], defective hematopoiesis and vasculogenesis [28], and delayed wound healing [31]. Hence, partial blockading of TGF-*β* may block only its antifibrotic effect and reduce the occurrence of other side effects. Studies have suggested that CDA1 (also known as TSPYL2, TSPX, Se20-4, NP79, CINAP, and DENTT) has a synergistic effect with TGF-*β*, which activates the TGF-*β* signaling pathway and promotes the occurrence and development of renal fibrosis in DN [32, 33]. However, the mechanism by which CDA1 enhances the TGF-*β* signaling pathway is not yet clear. CDA1 is not an essential component of the TGF-*β* signaling pathway, and TGF-*β* signaling cannot be completely blocked by the deletion of functional CDA1. Healthy CDA1 KO mice show normal growth and reproduction without abnormal phenotype [33], but blockade of CDA1 does not affect any essential processes mediated by TGF-*β* signaling, such as wound healing is unknown. Therefore, targeting CDA1 is a potential new strategy to delay the development of DN. It is worth mentioning that a prototype peptide inhibitor of CDA1 can safely and efficiently delay key renal parameters associated with diabetic-induced renal fibrosis in experimental animal models. However, it remains unclear whether this kind of research strategy can be reproduced in humans to delay the progression of DN.

## 2. Overview on CDA1

**Figure 1:**
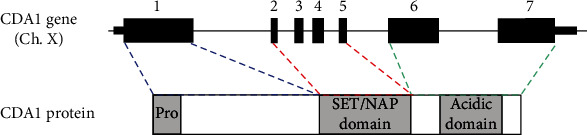
The gene and protein structure of CDA1. The CDA1 gene contains 7 exons, exon 1 encodes the N-terminal proline-rich domain, exons 2-5 encode the SET/NAP domain, and exons 6 and 7 encode the C-terminal acid domain.

**Figure 2:**
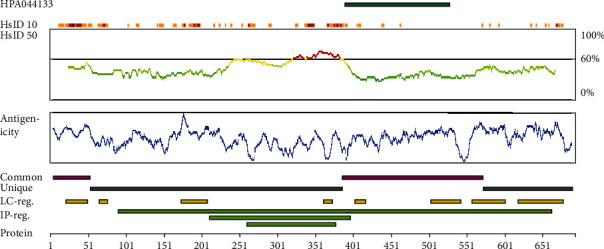
Antigenicity of CDA1. At the top of the diagram, the green bar shows the position of the antigen, with a length of 138 aa, located between 390 and 527 aa (This picture comes from The Human Protein Atlas (https://www.proteinatlas.org/)).

**Figure 3:**
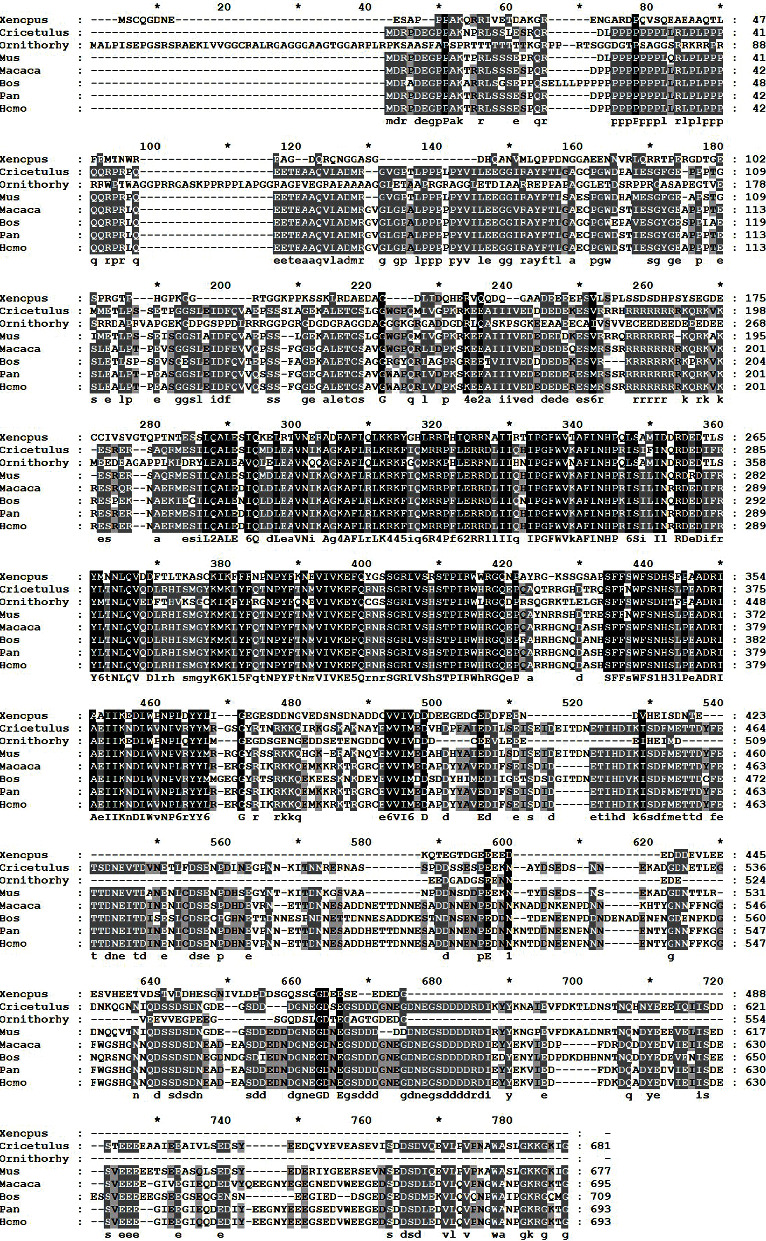
Multiple alignments of CDA1 between different species. Comparison of the amino acid sequence of CDA1 in humans and other species such as *Xenopus*, *Cricetulus*, *Ornithorhy*, *Mus*, *Macaca*, *Bos*, and *Pan*. In this alignment, the amino acids of other species are all identical to humans represented by black shadowing, and those of other species are, respectively, identical to humans represented by gray shadowing.

Chai et al. [34, 35] identified a novel protein in the serum of a patient with discoid lupus erythematosus. They named it CDA1 (cell division autoantigen 1). CDA1 is encoded by TSPYL2 (testis-specific Y-encoded-like protein 2) on the X chromosome. Its cDNA is composed of 2,808 base pairs, of which 2,079 base pairs of open reading frame encode 693 amino acids (aa) with a predicted molecular polypeptide of 79.43 KD and a pI of 4.26. The structure of CDA1 is shown in Figure 1. The antigen information of CDA1 shows that its antigen position is between 390 and 527 aa, with a length of 138 aa (Figure 2). Also, the amino acid sequences of CDA1 in humans and other species have been compared to each other, indicating that CDA1 is highly conserved among different species (Figure 3). Research from the Chai group suggests that CDA1 localizes in the nucleus of HeLa cells. However, since CDA1 has been detected in the cytoplasm, nucleus, or certain tissues of both adult mice and monkeys, it may shuttle between the cytoplasm and the nucleus [36, 37]. Biochemical studies show that CDA1 is a phosphorylated protein with multiple phosphorylation sites [34].

**Figure 4:**
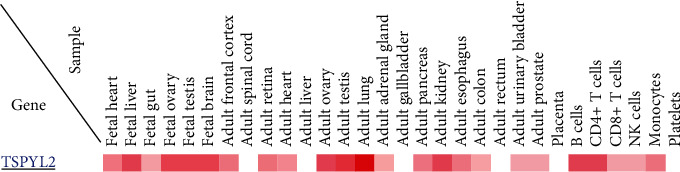
The CDA1 gene expression in different tissue and cells. CDA1 expression is highest in the brain and testis, followed by the ovary, adrenal gland, prostate, lungs, kidneys, and breasts, and is very low in the liver and pancreas (This picture is from Human Proteome Map (http://www.humanproteomemap.org/)).

Ozbun et al. [36, 37] found that CDA1 expresses in several tissues of the adult monkey and mouse (Figure 4). To further clarify the distribution and localization of CDA1 in humans, they observed its expression in adult monkey tissues by RT-PCR, Western blot, Northern blot analysis, and immunohistochemical staining and found that its highest expression in the brain and testis is followed by the adrenal gland, ovary, prostate, lung, and mammary gland. In addition, the level of CDA1 expression in the colon, kidney, lung, stomach, pancreas, and intestine is lower than that in the adrenal gland, cerebral cortex, and ovary. In adult monkey and developing mouse kidney, CDA1 expresses in the renal cortex and medullary tubules, but not in the glomeruli [36, 37]. Conversely, it is worth noting that the other two references reported that CDA1 not only expressed in glomerular podocytes in spontaneously hypertensive rats (SHRs) kidney and healthy human kidney but also suggested that CDA1 expression was significantly increased in human renal biopsy samples from both individuals with diabetic kidney disease and nondiabetic sclerotic renal disease [32, 33]. However, we still do not know why the CDA1 expression is different in the glomeruli with normal circumstances and diabetic condition. Moreover, CDA1 is also detectable in epithelial cells, fibroblasts, endothelial cells, smooth muscle cells, and chondrocytes in the monkey, and its expression is observed in many neurons including the peripheral and parasympathetic nervous systems of the central nervous system [36].

## 3. Function of CDA1

**Figure 5:**
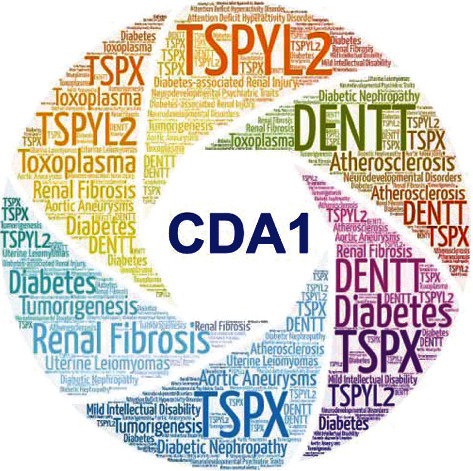
Summary of CDA1 function. CDA1, also known as TSPYL2, TSPX, DENTT, CINAP, Se20-4, and NP79, plays a positive or negative regulatory role in a variety of diseases such as various tumors, diabetes, DN, and atherosclerosis.

CDA1 exerts a variety of physiological functions, such as inhibiting tumorigenesis, inhibiting cell growth and proliferation, functioning as a cutaneous T-cell lymphoma specific antigen, maintaining normal function of the human heart, and promoting brain development (Figure 5).

### 3.1. Role in Tumorigenesis

CDA1 is widely expressed in normal tissues, but is downregulated in various types of cancer, including lung cancer [38], glioma [39], liver cancer [40], and prostate cancer [41]. Delbridge et al. [42] searched for a source of the TSPY gene associated with the gonadoblastoma formation (GBY) factor, a transcribed gene homologous to TSPY on the X chromosome of human and mouse. This source was identified and named as TSPX. The Y-localized TSPY and its X-homologue TSPX derived from the same ancestral genes but act as a protooncogene and a tumor suppressor gene, respectively. The C-terminal acidic domain (CAD) of TSPX is vital for the tumor suppressor function but is not contained in TSPY [43]. Tao et al. [44] have shown that TSPX serves a vital role in the maintenance of G1 checkpoint upon DNA damage. TSPX is also required for the induction of p21 transcription while the loss of TSPX results in cell cycle defect in the case of DNA damage. In addition, the CAD of TSPX also inhibits cyclin B1/CDK1 phosphorylation activity and performs a normative function in regulating cell-cycle progression at the G2/M stage [45]. Recent studies have also shown that TSPX is an important X-linked tumor suppressor gene in prostate cancer. Overexpression of TSPX could significantly inhibit cell proliferation and induce cell death in a prostate cancer cell line LNCaP. TSPX overexpression downregulated multiple oncogenes in a CAD-dependent manner and upregulated many tumor suppressors in a CAD-independent manner [41]. Besides, TSPX could interact with androgen receptors (AR) and inhibit the AR transactivation of target genes in a CAD-dependent manner [46]. Because androgen and AR play a fundamental role in the development of prostate cancer, TSPX might act as a modular for androgen and AR activities in the prostate. These results suggested that TSPX plays an important role in the initiation and progression of prostate cancer, and its CAD is essential for tumor suppressor functions.

Ozbun [38] has reported the discovery of a new gene in the TGF-*β*1 responsive epithelial non-small-cell lung cancer (NSCLC) cell line NCI-H727 and is named differentially expressed nucleolar TGF-*β*1 target gene (DENTT). These findings suggest that this new gene may be a target gene for TGF-*β* 1-mediated response, which is closely related to tumorigenesis, especially lung cancer. A subsequent study reported that messenger RNA (mRNA) and protein levels of DENTT were significantly downregulated in human lung tumors, human lung cancer cell lines, and mouse lung tumor models, and overexpression of DENTT significantly suppressed cell growth and clonogenic potential in lung and breast cell lines. In addition, DENTT mRNA and protein levels were elevated after ectopic expression of DENTT or treatment with TGF-*β*1 in lung cancer cells. In summary, their results suggested that DENTT played a suppressor role in tumor development [47]. Magni [48] has reported that CDA1 is a tumor suppressor and plays a pivotal role in regulating cell growth and DNA damage response. It is well known that P53 is an important tumor suppressor gene in humans, and its deletion or mutation increases the susceptibility of tumors. Upon DNA damage, P53 promotes transcription of specific genes, leading to cell cycle arrest, apoptosis, or senescence. P53 is a target protein of lysine acetyltransferase P300, and nicotinamide adenine dinucleotide- (NAD-) dependent deacetylase SIRT1 negatively regulates the activity of p300 activity toward p53, making P53 deacetylated and thus preventing P53-induced apoptosis. When DNA damage occurs, CDA1 inhibits SIRT1 to promote p53 acetylation and initiate p53-dependent cell death, thereby inhibiting tumorigenesis.

Chai et al. [34, 35] reported that overexpression of CDA1 in HeLa cells arrests cell growth and inhibits DNA synthesis. They found that the mutation of two CDK consensus phosphorylation sites abolished the function of CDA1 inhibited cell growth. This finding indicates that CDA1 acts a negative regulator of cell growth. Further research suggested that CDA1 plays an antiproliferation role by activating p53 and MEK/ERK1/2 MAPK pathway to upregulate p21Waf1/CIP1 transcription [49].

SPYL2/REST complex has been found to have an important inhibitory effect on epithelial cancers, and its antiproliferative effect is achieved by activating and enhancing TGF-*β* signaling. Epping et al. [50] reported that CDA1 is a hugely important part of the REST/NRSF transcriptional complex and plays a role in tumor inhibition by interacting with REST to enhance TGF- *β* signaling.

### 3.2. As a Cutaneous T-Cell Lymphoma-Specific Antigen

Eichmuller et al. [51] found a new gene, named as se20-4 (accession numbers: AF273046), in the serum and tissues of patients with cutaneous T-cell lymphoma (CTCL) when using SEREX (serological identification of recombinantly expressed genes) method to identify cutaneous T lymphoma-specific antigens. This identified antigen se20-4 showed high homology to the already known gene CDA1; they were the same gene (GenBank database accession numbers: AB015345). Further studies have confirmed that se20-4 is also present in tumor tissues and cell lines such as melanoma, leukemia [52], and lymphoma [53] and is a nuclear targeting protein.

### 3.3. As Differentially Expression Genes in Human Heart Tissues

Sun et al. [54] identified three novel genes in the genes differentially expressed between normal human and congenital heart defect (CHD) heart tissues by RNA arbitrarily primed-PCR (RAP-PCR) and RT-PCR, including NP79 (coding for a nuclear protein of 79KD), which is likely to be related to the normal function of the human heart. NP79 (accession numbers: AF273046) is identical to *H. sapiens* CDA1 mRNA and is also a clone for cutaneous T-cell lymphoma-associated antigen isolated from a normal testis cDNA library. Since NP79 is highly expressed in the adult heart, it is speculated that it plays a role in maintaining the normal function of the human heart.

### 3.4. Role in Brain Development

Wang et al. [55] identified a new gene named CINAP (CASK-Interacting Nucleosome Assembly Protein), from an adult rat brain library in a yeast two-hybrid screen using the guanylate kinase (GK) domain of CASK as baits. CINAP contains the NAP domain of the NAP/SET/TSPY family common characteristic structure. Thus, CINAP belongs to this family and is a homologous mouse gene of CDA1. CDA1 was found to be a nuclear protein mainly present in the cerebral cortex. CDA1, Tbr-1 (an essential transcription factor in cerebral cortex development), and CASK (acts as a coactivator for Tbr-1) form a complex in the nucleus of neurons to regulate in response to neuronal synaptic activity and chromatin assembly [55, 56]. Several pieces of research have shown that mutations or deletion of CDA1 are associated with varying degrees of intellectual disability [57–59], suggesting that CDA1 may play an important role in learning and behavior. According to a study, CDA1 KO mice showed higher locomotor and exploratory activities compared to their wild-type littermates, but the molecular mechanism is unclear [60]. Glutamate pathology is involved in neurodevelopmental conditions, and CDA1 regulates the expression of genes encoding glutamate receptors. CDA1 KO mice may have neurodevelopmental and behavioral abnormalities due to the disruption of glutamate signaling, mainly manifested as sensorimotor gating impairment, mild hyperactivity, and hypersensitivity to the dopamine agonist amphetamine [61]. Subsequently, the study found that compared with wild type littermates, the expression of H3K27me3 (a key histone modification important for brain development and neuronal function) in the hippocampus of CDA1 KO mice was upregulated. In addition, the expression of H3K27 methyltransferase enhancer of zeste 2 (EZH2) target genes important for neuron function was downregulated. Chromatin immunoprecipitation (ChIP) reveals that hemagglutinin-tagged CDA1 coexist in EZH2 in the target promoter of human neuroblastoma cells. These findings indicated that CDA1 interacted with EZH2 and enhanced the expression of H3K27me3-labeled neuronal genes to achieve normal neuronal maturation and function [62]. All the above results indicate that CDA1 is a critical gene for neurodevelopment. In addition, crosstalk between CDA1 and TGF-*β* signaling has been described [35], signaling by the TGF-*β* family playing a central role in many aspects of nervous system development and function [63], suggested that TGF-*β*-CDA1 signaling pathway could be essential in regulating neurogenesis and neurological function.

## 4. CDA1 and Renal Fibrosis in DN

It has been found that CDA1 plays a positive or negative regulatory role in a large number of different diseases (Table 1), and it can play a nonnegligible role in DN renal fibrosis by enhancing the TGF-*β* signaling pathway, but its specific mechanism is not yet clear.

### 4.1. CDA1 Promotes the Occurrence and Development of Renal Fibrosis in DN

Chai's team [32] has found that CDA1 expression level in the kidneys of diabetic animal models was elevated. In addition, compared with healthy kidneys, the expression of CDA1 was significantly increased in the kidneys of patients with DN and patients with nondiabetic sclerotic renal disease [33]. *In vivo* assays, they used spontaneously hypertensive rats (SHRs) and the ApoE^−/−^ mouse as a model of DN. The results showed that the mRNA levels of TGF-*β*, TGF-*β* type I receptor (T*β*RI), TGF-*β* type II receptor (T*β*RII), connective tissue growth factor (CTGF), collagen I (COL1), III (COL3), IV (COL4), and fibronectin (FN) were significantly increased compared to their nondiabetic controls. *In vitro* assays forced expression of CDA1 in HK-2 cells resulted in TGF-*β* signaling pathway activation by upregulation of these genes, while knockdown of CDA1 in HK-2 cells significantly reduced TGF-*β* signaling and downregulated the expression of these genes. Moreover, recombined TGF-*β* treatment of CDA1-overexpressing HK-2 cells resulted in further activation of TGF-*β* signaling, and its downstream of ECM genes such as COL1 and COL3, but CDA1 knockdown effectively blocked TGF-*β*-stimulated expression of collagen genes. These results suggested that the molecular synergy between CDA1 and TGF-*β* underpin TGF-*β* signaling and its target gene expression [32]. Furthermore, compared with CDA1 wild-type (WT) mice, the expression of TGF-*β*, T*β*RI, T*β*RII, and key target genes of TGF-*β* associated with diabetes including CTGF, *α* smooth muscle actin (*α*-SMA), COL1, COL3, and COL4, as well as monocyte chemoattractant protein-1(MCP-1) and vascular cell adhesion molecule 1(VCAM-1) and FN were significantly attenuated in diabetic CDA1 KO mice. CDA1 deletion reduced the glomerular and tubulointerstitial injury indexes in CDA1/ApoE gene dKO mice. Deletion of CDA1 disrupted TGF-*β* signaling, failing TGF-*β* to stimulate the expression of its target genes such as COL1 and COL3 in primary kidney cells isolated from CDA1 WT and KO mice. CDA1 deficiency in diabetic mice attenuated TGF-*β* signaling, leading to a reduction in ECM accumulation in the kidney [33]. These results suggest that CDA1 plays a vital role in the development of DN. Thus CDA1 may increase the production of ECM protein in the kidney of diabetic patients by enhancing TGF-*β* signaling, thereby promoting the development of renal fibrosis of DN. Studies have also shown that CDA1 appears to be a significant molecule not only in DN but also in other diabetic vascular complications such as atherosclerosis [68]. Li et al. [66] indicated that CDA1 plays a key role in the protective effect of diabetes on aneurysms. However, the specific mechanism by which CDA1 enhances the TGF-*β* signaling pathway and promotes renal fibrosis in DN deserves further investigation.

### 4.2. The Possible Mechanism of CDA1 Promoting Renal Fibrosis in DN through the TGF-*β* Signaling Pathway

**Figure 6:**
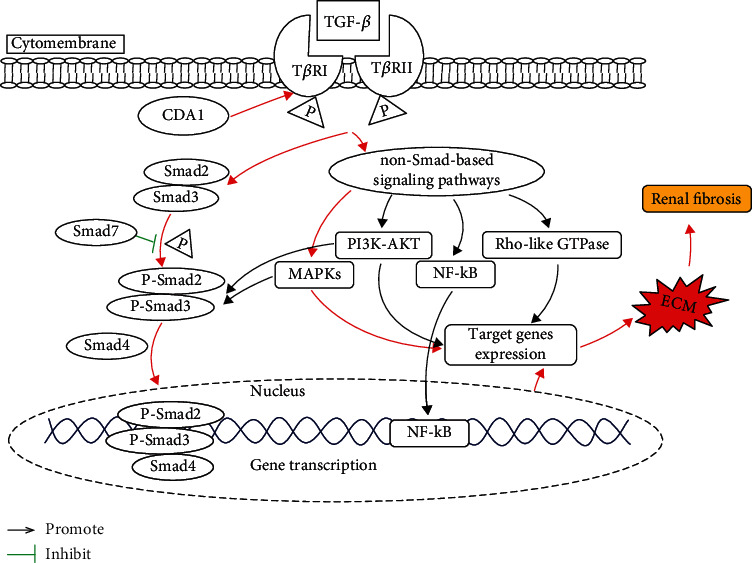
The possible mechanism of crosstalk between CDA1 and TGF-*β* promoting renal fibrosis in DN. The canonical TGF-*β* signaling pathway and MAPK signaling pathway marked as red arrow have been demonstrated to be associated with CDA1 expression in diabetic nephropathy, while pathways marked as black related with CDA1 expression remain unknow.

Downstream of the TGF-*β* signaling pathway includes canonical (Smad-based) and noncanonical (non-Smad-based) signaling pathways. The canonical pathway is mainly through Smads signaling pathway. Non-Smad-based pathways include MAPKs (including ERK, p38, and JNK), NF-*κ*B, PI3K-AKT, and Rho-like GTPase [69]. CDA1 may be involved in these pathways of TGF-*β* to promote renal fibrosis in DN (Figure 6).

#### 4.2.1. Through the TGF-*β*/Smad Signaling Pathway

TGF-*β* is a bioactive polypeptide and a member of the transforming growth factor superfamily. Its three isoforms including TGF-*β*1, TGF-*β*2, and TGF-*β*3 have been successively cloned in mammals [70], mainly through the mechanism of autocrine or paracrine to reach the corresponding target organs to perform their multiple functions [71]. TGF-*β* binds to and gathers together with serine/threonine kinase receptors (mainly including two subtypes T*β*RI and T*β*RII with similar structures) on the cell membrane to initiate signaling [72]. The subtype of TGF-*β* in the kidneys is mainly TGF-*β*1, with studies suggesting that TGF-*β*1 or its signaling downstream signaling pathway is the major pathogenic factor leading to onset or progression of forms of renal fibrosis [22]. The main mechanisms of TGF-*β*1 inducing renal fibrosis include (1) directly inducing the synthesis of ECM, including COL1 and FN, from the transcription level [73]; (2) inducing the imbalance of matrix metalloproteinases (MMPs) and tissue inhibitor of metalloproteinase (TIMPs) to inhibit the degradation of ECM [74]; (3) directly affecting renal intrinsic cells: it can induce mesangial cell proliferation and collagen secretion, promote epithelial and podocyte damage, and aggravate inflammation and secondary fibrosis, etc. [75]; (4) promoting the transdifferentiation and proliferation of myofibroblasts from various sources such as pericyte, fibroblast, epithelial cells, and macrophages, as well as mediating fibrosis responses [76]. Usually, the downstream effector transcription factor of TGF-*β* signaling pathway is Smad. They exist in the cytoplasm, with their main function to transduce TGF-*β* signals from the cell membrane into the nucleus. The sequential phosphorylation reaction process sees TGF-*β* activating Smad. T*β*RII of TGF-*β* binds to the ligand and phosphorylates T*β*RI, which in turn phosphorylates Smad2/3 (membrane-receptor-activated Smad, R-Smad), then undergoes homotrimerization and formation of heteromeric complexes with the Smad4 (comediator Smad, co-Smad). This complex moves into the nucleus and interacts with various transcription factors to regulate the transcription of target genes [77]. Activated T*β*RI phosphorylates R-Smad, initiating the TGF-*β* signaling pathway as well as activating Smad7 (inhibitory Smad, I-Smad). Smad2 and Smad3 are two main downstream regulators of TGF-*β* 1-mediated tissue fibrosis, and Smad7 acts as an inhibitory regulator of the TGF-*β*1/Smad pathway to prevent TGF-*β* 1-mediated fibrosis [78]. Several previous studies have proven that renal fibrosis can be attenuated by regulating the TGF*β*/Smad3 signaling pathway [79–82]. Chai et al. found that CDA1 enhances TGF-*β* signaling by regulating T*β*RI and increases phosphorylation of Smad3 in HK-2 cells, primary kidney cells from CDA1 WT mice, tubular cells, and glomerular podocytes in ApoE KO and CDA1/ApoE dKO mice, which result in accumulation of ECM in the kidney, resulting in the development of DN [32, 33]. Therefore, CDA1 may promote the development of diabetic renal fibrosis through the TGF-*β*/Smads signaling pathway.

#### 4.2.2. Through TGF-*β*/MAPK Signaling Pathway

Mitogen-activated protein kinases (MAPKs) are important non-Smad-dependent downstream signaling pathways of TGF-*β*. Studies have found that MPPK's family members including extracellular signal-regulated kinase (ERK), mitogen-activated protein kinase (p38), and C-Jun N-terminal kinases (JNK) were significantly involved in renal fibrosis. Studies have demonstrated that TGF-*β*1 could activate MAPKs, then induced apoptosis in renal podocytes thereby accelerating DN progression [83–85]. Lakshmanan et al. [86] found that p38 MAPK, ERK, and JNK were activated in streptozotocin- (STZ-) induced diabetic kidney disease in mice, and these protein expressions were upregulated. Furthermore, crosstalk between ERK, JNK, and p38 MAPKs and Smad signaling pathways could synergistically enhance the expression of TGF-*β*-induced profibrotic genes in various types of renal cells to promote renal fibrosis [87–90]. It has been found that some drugs can reverse renal fibrosis by inhibiting the MAPK pathway [91, 92]. Studies have suggested that CDA1 could increase the phosphorylation of ERK/MAPK in animal models of DN and increase the accumulation of ECM in the kidney, leading to the development of DN. Conversely, CDA1 deletion attenuated TGF-*β* signaling pathway by attenuating phosphorylation of Smad3 and ERK/MAPK in primary kidney cells from CDA1 WT mice, thereby reducing renal ECM accumulation [32, 33]. As a result, CDA1 may promote the development of diabetic renal fibrosis through the TGF-*β*/MAPKs signaling pathway.

#### 4.2.3. Through TGF-*β*/NF-*κ*B Signaling Pathway

Nuclear factor kB (NF KB) is a vital transcription factor, which not only mediates various immune and inflammatory reactions but also participates in a wide range of biological processes, including cell proliferation, differentiation, autophagy, and senescence [93]. TGF-*β* can activate NF-*κ*B and mediate transcriptional activation of TGF-*β* target genes in a variety of cell types [93]. It has been found that NF-*κ*B signaling can participate in the occurrence of renal fibrosis. In the priming phase of renal fibrosis, direct renal tubular epithelial cell injury or cellular stimuli triggers the production of various proinflammatory molecules driven by activation of NF-*κ*B signaling, leading to the recruitment of inflammatory cells, thus mediates inflammatory response and promotes the occurrence of renal fibrosis [94, 95]. NF-KB signaling pathway could mediate hypoxia-induced inflammatory responses, ECN accumulation, and oxidative stress leading to renal fibrosis [96]. These results suggest that NF-*κ*B signaling pathway plays a crucial role in the development of renal fibrosis. CDA1 can promote renal fibrosis by regulating TGF-*β* signaling, and NF-*κ*B can serve as the downstream signaling pathway of TGF-*β*. Therefore, it is speculated that CDA1 can promote the progression of diabetic nephropathy through the TGF-*β*/NF-*κ*B signaling pathway. However, there is no direct evidence that CDA1 is involved in renal fibrosis of DN through the TGF-*β*/NF-*κ*B signaling pathway.

#### 4.2.4. Through TGF-*β*/PI3K-AKT Signaling Pathway

TGF-*β* and phosphoinositide 3-kinase/protein kinase B (PI3K-Akt) signaling pathway controls a variety of cellular responses, including glucose homeostasis, cell proliferation, apoptosis, migration, and survival [93, 97]. Studies have reported that extensive crosstalk between TGF-*β* and the PI3K/AKT signaling pathway plays a key role in tumor progression. In the early stages of cancer, the activated PI3K/AKT pathway antagonizes TGF-*β*/Smad induced the cytostatic or apoptosis response, whereas, in advanced cancers, both two pathways synergistically cooperate in promoting the invasiveness of cancer cells [97]. The PI3K/AKT signaling pathway not only regulates the occurrence of tumors but is also involved in the regulation of renal fibrosis. Runyan et al. [98] found that the crosstalk between Smad and PI3K/Akt pathway may contribute to TGF-*β*-induced glomerular matrix accumulation. PI3K-AKT signaling pathway could mediate hypoxia-induced fibroblast activation, collagen synthesis, and EMT regulation leading to renal fibrosis [91]. Several studies have confirmed that inhibition of the PI3K/AKT signaling pathway can alleviate renal interstitial fibrosis [99–101]. These results suggest that the PI3K/AKT signaling pathway crosstalk with the TGF-*β* signaling pathway plays a pivotal role in the progression of tumors and renal interstitial fibrosis. CDA1 can regulate renal fibrosis and inhibit tumorigenesis through the TGF-*β* signaling pathway. Therefore, it is speculated that CDA1 can promote the progression of DN through the TGF-*β*/PI3K-AKT signaling pathway. Direct evidence of CDA1 participating in the development of renal fibrosis via the TGF-*β*/PI3K-AKT signaling pathway is still lacking. This question needs to be further clarified in subsequent studies.

#### 4.2.5. Through TGF-*β*/Rho-Like GTPases Signaling Pathway

Rho GTPases are members of the Ras superfamily, while the three best characterized mammalian members are Rho, Rac, and Cdc42 [102]. ROCK, also known as Rho-associated kinase, belongs to the serine/threonine-protein kinase. ROCK is the most important and characteristic Rho downstream target effector molecule and is widely expressed in human body. It has many functions such as regulating cell contraction, motility, cell division, adhesion, and proliferation, and it participates in the occurrence and development of a variety of diseases [103]. Studies have demonstrated that DN is associated with the activation of the Rho/ROCK signaling pathway [104]. The application of ROCK inhibitors can delay the development of renal interstitial fibrosis [105–108]. Furthermore, TGF-*β* has been discovered to induce activation of Rho, Rac, and Cdc42 in different cell systems. However, most studies have concentrated on the role of RhoA and its effector kinase ROCK in TGF-*β*-induced epithelial-to-mesenchymal transdifferentiation (EMT) [109]. It was found that TGF-*β* rapidly activates RhoA-dependent signaling pathways in epithelial cells and promotes the occurrence of EMT by regulating cytoskeletal remodeling and activating SMA promoter [110, 111]. CDA1 regulates the TGF-*β* signaling pathway, while Rho-like GTPase is a Smad-independent downstream signaling pathway of TGF-*β*. Thus, CDA1 may promote the development of diabetic renal fibrosis through TGF-*β*/Rho-like GTPase signaling pathway. However, there is currently no evidence that CDA1 promotes the development of renal fibrosis of DN through the TGF-*β*/Rho-like signaling pathway.

### 4.3. Current Method for Targeting CDA1 to Treat Renal Fibrosis of DN

Targeting CDA1 has been demonstrated to attenuate renal fibrosis-related genes expression by regulating TGF-*β* signaling in several animal models and in *in vitro* studies. Chai's team [33] generated CDA1 KO mice by genetic deletion of *Tspyl2*. They found that CDA1 KO mice had no significant effect on animal health, while no obvious abnormal phenotypes were found during reproduction. Genetic deletion of CDA1 was found to significantly reduce the expression of diabetes-associated renal matrix accumulation and profibrosis genes both in vivo and in vitro [32, 33], confirming that CDA1 is an important and effective potential target in DN.

Recently, Chai et al. [67] isolated and identified a novel protein interacting with CDA1 from a human testis testicular cDNA library using yeast two-hybridization screening, which was called CDA1 binding protein 1 (CDA1BP1). They found that CDA1 and CDA1BP1 were expressed in human and mouse kidneys, as well as human renal tubular HK-2 cells. CDA1BP1 binds to CDA1 and enhances TGF-*β* signaling to exert its profibrotic effect. They utilized the model of diabetic by using STZ-induced male ApoE KO, WT, and CDA1BP1 KO mice, all with the background of C57BL6. The expression level of COL1, COL3, FN, and tumor necrosis factor-*α* (TNF-*α*) were significantly reduced in diabetic CDA1BP1 KO mice renal. Also, CDA1BP1 siRNA knockdown reduced the level of COL1 and COL3 in HK-2 cells with or without TGF-*β* treatment. Furthermore, Chai's team generated a hybrid peptide inhibitor CHA-061, which contains a short CDA1BP1 sequence with an ability to bind to CDA1, and a cell penetrating peptide [112, 113]. CHA-061 treatment significantly attenuated or blocked the expression of diabetes-associated profibrotic genes TGF-*β*1, TGF-*β*2, T*β*RI, T*β*RII, and CTGF, sclerotic genes such as COL1, COL3, COL4, FN, and MMP2, proinflammatory genes such as TNF-*α*, C-reactive protein (CRP), MCP-1, intercellular adhesion molecules-1 (ICAM1), and vascular cell adhesion molecule (VCAM1) in the kidneys. It also reduced renal ECM accumulation and glomerular injury index. No significant side effects were observed during the study. These studies suggested that CDA1/CDA1BP1 axis targeting by genetic and pharmacological approaches is a safe and effective method to attenuate pathological hallmarks and to inhibit renal fibrosis of experimental DN.

## 5. Conclusion

CDA1 is distributed in various tissues of the human body exerting various biological functions, such as inhibiting cell growth and inhibiting tumor proliferation. It also plays a crucial role in renal fibrosis of DN. Quite a few studies have shown that CDA1 can enhance renal and vascular TGF-*β* signaling and promote the development of renal fibrosis in DN. However, the specific mechanism remains unclear. CDA1 may act on T*β*RI, induce Smad phosphorylation, and activate the ERK MAPK pathway to promote renal fibrosis and atheroma formation in DN. No abnormal phenotype was observed in CDA1 KO DN mice, which were able to develop and grow normally, with only mild kidney damage. Therefore, CDA1 may be a safe and effective therapeutic target for delaying DN and other mediated renal fibrosis induced by TGF-*β*. Recently, prototype peptide inhibitors of CDA1 have been developed with their safety and efficacy having been verified genetically and pharmacologically. In the future, more drugs need to be developed to target CDA1 in the treatment of DN. In addition, due to the complexity of the TGF-*β* signaling pathway, whether CDA1 enhances TGF-*β* signaling through other mechanisms requires further validation in future studies.

## Figures and Tables

**Table 1:** Studies of CDA1 in different diseases.

Disease type	Strategy	Regulation	Research object	Pathway/process	Function	References
Lung cancer	Transfection	CDA1↓	(1) *In vivo*: mice; (2) *in vitro:* A549 cells; (3) human lung tumor	Cyclin B-CDK1↓; TGF-*β*1↓	↓Cell growth; ↓cell migration	[47]
Breast cancer	Transfection	CDA1↓	(1) *In vivo*: mice; (2) *in vitro:* MCF-7 cells	Cyclin B-CDK1↓	↓Cell growth; ↓cell migration	[47]
Prostate cancer	Lentivirus	CDA1↓	(1) *In vitro:* LNCaP cells, PC3 cells, DU145 cells; (2) human prostate	Cyclin B-CDK1↓	↓Androgen receptor; ↓oncogenes; ↑tumor suppressors	[41]
Malignant glioma	Inhibitors	CDA1↓	(1) *In vitro*: T98G cells; (2) human glioblastoma, human multiforme, human glioma, human astrocytoma, human oligodendroglioma	Hypermethylation	↓Cell growth; ↓cell migration	[64]
Hepatocellular carcinoma	Transfection	CDA1↓	*In vitro:* 293T cells, HuH7 cells	Ubiquitin-proteasome ↑; hepatitis B viral protein (HBx)↓	↓Cell growth; ↓cell migration	[65]
Aortic aneurysms	Gene knockout	CDA1↓	(1) *In vivo*: mice; (2) human abdominal aortic aneurysms	TGF-*β*1↓	↓Aneurysm formation in human; ↓aneurysm severity in mice	[66]
Diabetic nephropathy	Gene knockout; adenoviral	CDA1↓	(1) *In vivo*: mice; (2) *in vitro:* HK-2 cells; (3) human renal biopsy	TGF-*β* ↓	↓Renal fibration	[32, 33, 67]
Diabetic atherosclerosis	Gene knockdown adenoviral	CDA1↓	(1) *In vivo*: mice; (2) *in vitro:* vascular smooth muscle cells	CDA1↓	↓Atherosclerosis	[68]
Attention deficit hyperactivity disorder	Gene knockdown	TSPYL2↓	*In vivo*: mice	Glutamate receptors↓	↑Activity; ↓prepulse inhibition	[61]
Neurodevelopmental psychiatric disorders	Gene knockdown	TSPYL2↓	(1) *In vivo*: mice; (2) *in vitro:* HEK293 cells	Glutamate↓; GluN2A↓, GluN2B↓; BDNF↓, Egr3↓, Grin2c↓	↓Learning and behavior	[59, 62]
Intellectual disability	Genetic studies	Mutations in SPYL2	Mild nonsyndromic ID	Synaptic signaling↓	↓Memory skills and language development	[57, 58]

↓: decrease; ↑: increase.
